# Mitochondria in precision medicine; linking bioenergetics and metabolomics in platelets

**DOI:** 10.1016/j.redox.2019.101165

**Published:** 2019-03-10

**Authors:** Balu K. Chacko, Matthew R. Smith, Michelle S. Johnson, Gloria Benavides, Matilda L. Culp, Jyotsna Pilli, Sruti Shiva, Karan Uppal, Young-Mi Go, Dean P. Jones, Victor M. Darley-Usmar

**Affiliations:** a Mitochondrial Medicine Laboratory, Department of Pathology, University of Alabama at Birmingham, UK; b Clinical Biomarkers Laboratory, Division of Pulmonary, Allergy, and Critical Care Medicine, Emory School of Medicine, Atlanta, GA, USA; c Department of Pharmacology & Chemical Biology, Vascular Medicine Institute, Center for Metabolism & Mitochondrial Medicine, University of Pittsburgh, Pittsburgh, PA, USA

## Abstract

Mitochondria possess reserve bioenergetic capacity, supporting protection and resilience in the face of disease. Approaches are limited to understand factors that impact mitochondrial functional reserve in humans. We applied the mitochondrial stress test (MST) to platelets from healthy subjects and found correlations between energetic parameters and mitochondrial function. These parameters were not correlated with mitochondrial complex I-IV activities, however, suggesting that other factors affect mitochondrial bioenergetics and metabolism. Platelets from African American patients with sickle cell disease also differed from controls, further showing that other factors impact mitochondrial bioenergetics and metabolism. To test for correlations of platelet metabolites with energetic parameters, we performed an integrated analysis of metabolomics and MST parameters. Subsets of metabolites, including fatty acids and xenobiotics correlated with mitochondrial parameters. The results establish platelets as a platform to integrate bioenergetics and metabolism for analysis of mitochondrial function in precision medicine.

## Highlights

• Variations in platelet bioenergetics can be ascribed to the metabolic plasticity required for physiological metabolism.
• Bioenergetic parameters in normal subjects are related. These relationships deteriorate in Sickle Cell disease patients.
• Untargeted metabolomics reveals over 100 potential metabolites that underlie the mitochondrial stress test.
• Among the metabolites associated with platelet bioenergetic parameters are environmental toxins and natural products.

## Graphical abstract

**Figure:**
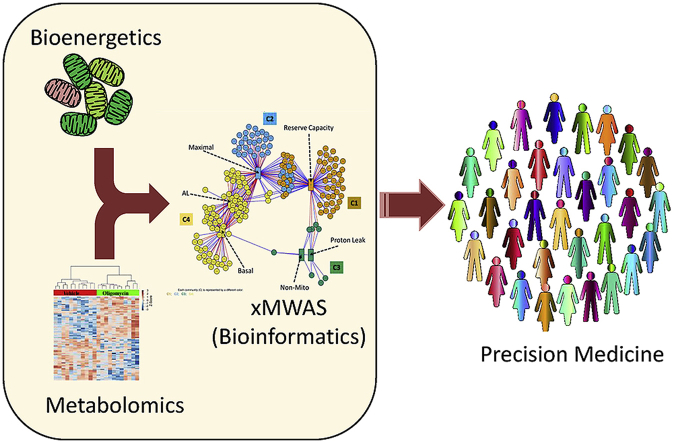

## 1 Introduction

The susceptibility to a broad range of diseases including diabetes and aging-related neurodegenerative pathologies such as Alzheimer's Disease have been shown to be linked to mitochondrial metabolism [[1], [2], [3], [4], [5], [6], [7]]. In addition to the potential direct contribution of dysfunctional bioenergetics to the mechanism of these diseases, the failure to maintain adequate mitochondrial quality to protect against oxidative or metabolic stress also appears to be important [8,9]. It becomes of interest, from a precision medicine perspective, to develop diagnostic and prognostic indices of bioenergetic health. For example, it has been shown that mitochondria-related biomarkers and metabolites can predict the clinical outcome for sepsis patients [[10], [11], [12]].

Interestingly, it is now becoming clear that platelets can serve as biomarkers for mitochondrial dysfunction [1]. Changes in platelet bioenergetics or mitochondrial function have been linked to sickle cell disease (SCD), asthma, Alzheimer's and Parkinson's disease [3,13,14]. Recent advances in the measurement of platelet bioenergetics allow the determination of parameters, which reflect metabolism in the intact platelet and assignment of oxygen consumption to ATP synthesis and the overall capacity of oxidative phosphorylation [2,15]. This assay, known as the mitochondrial stress test (MST), is based on the sequential addition of mitochondrial inhibitors and the concomitant measurement of oxygen consumption and extracellular acidification rates (OCR and ECAR) [9,16]. The recent application of this method using platelets, monocytes and lymphocytes to clinical samples has revealed the potential of the MST for diagnostic and prognostic translational research [1,2,7,13,14,17,18]. Platelet metabolism has a high level of metabolic plasticity with fuel utilization switching to fatty acids as a requirement for both oxidative phosphorylation and aggregation [[19], [20], [21]]. In the present study with intact human platelets, we show a significant variation in oxygen consumption rates for key parameters such as ATP-linked respiration between individuals. This potential variation in mitochondrial efficiency should also be reflected in the metabolome and potentially contribute to the susceptibility to stressors. For example, we have shown that platelets respond to the toxicity of the lipid peroxidation product 4-hydroxynonenal by stimulation of glycolysis but inhibition of the TCA cycle and mitochondrial function [22].

In platelets from patients with SCD, a pathology characterized by hemolytic anemia due to a mutation in the beta-globin gene of hemoglobin, the MST showed decreased basal and oligomycin-sensitive oxygen consumption, with no significant change in maximal capacity compared to platelets from healthy matched controls [14]. We demonstrated that this bioenergetic alteration was due to significant inhibition of complex V (ATP synthase) in SCD subjects, and resulted in increased membrane potential and oxidant production. Notably, this inhibition of platelet complex V was due to exposure of platelets to plasma free hemoglobin, which is increased by hemolysis. Additionally, the resulting mitochondrial oxidant production stimulates platelet thrombotic activation and susceptibility to aggregation [14]. This mechanism may underlie the increased basal platelet activation in SCD patients and contribute to the increased incidence of thrombotic disease in this population [[23], [24], [25]].

Our previous studies have suggested that the parameters revealed by the MST are inter-dependent [9]. The bioenergetic parameters measured by the MST reflect the ability of the platelet to provide the fuel necessary to drive oxidative phosphorylation. We have shown that in the platelet, there are contributions from glycolysis, fatty acids, and glutamine to the MST profile, which also varies with thrombin-dependent aggregation [19,20]. However, whether the capacity of the mitochondrial complexes that compose the oxidative phosphorylation pathway are limiting is not known. By developing an integrated assay in which both oxidative phosphorylation and bioenergetics in the intact platelet can be measured in parallel samples from the same individual donor we were able to address this question. It is expected that mitochondrial metabolism will vary between healthy individuals and will be reflected in both the metabolome and the MST which is a measure of the integration of the components of oxidative phosphorylation and cellular metabolism. In the present study, we tested these hypotheses using untargeted metabolomics in platelets from healthy donors and found that regulation of bioenergetic parameters derived from the MST are well correlated with over one hundred metabolites.

## 2 Materials and methods

### 2.1 Chemicals

All reagents were purchased from Sigma-Aldrich (St. Louis, MO, USA) unless otherwise specified. A mixture of internal standard stable isotopic chemicals [26,27] from Cambridge Isotope Laboratories, Inc. (Andover, Pennsylvania).

### 2.2 Platelet isolation

Platelets used for these studies were between day 6 and 8 after collection or freshly isolated as described previously [[14], [15], [16]]. Based on our previous studies platelets were isolated and assayed within 4–6 h of collection of the blood sample over which time the bioenergetic function is stable(15). Platelet concentrates from 11 to 13 individual donors were obtained from the University of Alabama at Birmingham blood bank at day 6–8 as described in Ref. [28] or isolated from fresh blood samples from healthy donors (85 samples) [15]. For the healthy donors a screening survey was used to select for healthy patients to ensure they were disease free at the time the blood sample was taken. Exclusion criteria were pregnancy, smoking, active diseases or surgical procedures within one month, medications (antibiotics, steroids, HIV medications, anti-depressants, anti-inflammatory drugs within 48 h, BMI over 40 etc.) or alcohol within 48 h of collection. The demographics for this group are reported in Table 1a. Collection and use of these samples were approved by the University of Alabama at Birmingham Institutional Review Board (Protocol #X110718014). Platelets from sickle cell disease (SCD) patients and healthy subjects (n = 35 in each group) were freshly isolated from blood collected after informed consent on Protocol# PRO08110422, which was approved by the University of Pittsburgh Institutional Review Board. All subjects were African American and between 25 and 45 years of age and the detailed demographics are reported in Table 1b. Subjects with SCD were homozygous SCD (HbSS) and in steady state. African American subjects had no known hemoglobinopathy. Subjects were excluded if they were on any anti-coagulant medication or had received a blood transfusion in the three months prior to blood draw. In brief, platelets were pelleted by centrifuging at 1500 g for 10 min then washed with PBS containing prostaglandin I_2_ (1 μg/ml) and the platelet number was determined by turbidimetry [29]. Platelet aggregation using the 96-well plate reader was measured as previously described [30].

**Table 1a:** Healthy subject demographics.

Characteristic	N = 85	(%)
Age (years):		
Median	33	
Range	18–64	
Gender:		
Female	43	(50.6)
Male	42	(49.4)
Race:		
Asian	19	(22.4)
African American	22	(25.9)
Caucasian	42	(49.4)
Hispanic	1	(1.2)
Unknown	1	(1.2)

**Table 1b:** Demographics of sickle cell and healthy African American subjects.

	African American Controls (n = 35)	Sickle Cell (n = 35)
Age Range (median)	26-45 [37]	25-45 [36]
Gender (Male: Female)	17: 18	15: 20

### 2.3 Treatment and assessment of platelet bioenergetics and mitochondrial function

The 96-well format Seahorse extracellular flux analyzer (Seahorse Bioscience, MA, USA) was used to measure bioenergetics [16]. Platelets were diluted to a concentration of 1 × 10^7^ in XF DMEM assay buffer (DMEM with 1 mM pyruvate, 5.5 mM d-glucose, 4 mM l-glutamine, pH 7.4) and were seeded onto Cell-Tak coated XF96 microplates and mitochondrial stress test was performed as described [14,31]. The mitochondrial complex assay is performed using Plasma Membrane Permeabilizer (PMP) with an injection of respiratory substrates with ADP or FCCP [32].

### 2.4 High-resolution metabolomics (HRM)

Untargeted metabolomics was performed using previously established HRM methods [[33], [34], [35], [36]]. Washed platelets were diluted to a concentration of 100 × 10^6^/well in 0.75 ml DMEM assay buffer (DMEM with 1 mM pyruvate, 5.5 mM glucose, 4 mM glutamine, pH 7.4) and treated with oligomycin (1 μg/ml) for 30 min at 37 °C in a non-CO_2_ humidified incubator in 6-well plates (9 cm^2^/well). Platelets were then washed with cold PBS and the proteins precipitated using acetonitrile (50 μl) containing a mixture of stable isotope-labeled internal standard [26,27]. Pooled platelets (300 × 10^6^ platelets from 3 wells) in 150 μl of acetonitrile containing internal standard were incubated on ice for 30 min, metabolic extracts were centrifuged to remove proteins, randomized, and 10 μL aliquots were analyzed with three technical replicates using reverse-phase C_18_ liquid chromatography (Targa C_18_ 2.1 mm × 50 mm x 2.6μm, Higgins Analytical) combined with a High Field Q-Exactive mass spectrometer (Thermo Fisher). Mass spectral detection completed in negative mode electrospray ionization (ESI) at 120,000 (FHWM) resolution over a mass-to-charge ratio (*m/z*) range of 85–1250. A quality control pooled reference plasma sample (Q-Std3) was included at the beginning and end of each batch of 25 samples for quality control and quality assurance [37]. Raw data files were extracted using apLCMSv6.3.3 [38] with xMSanalyzerv2.0.7 [39], followed by batch correction with ComBat [40]. Uniquely detected ions consisted of *m/z*, retention time (RT) and ion abundance, referred to as metabolic features.

### 2.5 Data processing and metabolic feature selection

Prior to data analysis, triplicate injections were averaged and only *m/z* features with at least 80% non-missing values in either of the groups and more than 40% non-missing values across all samples were retained. After filtering based on missing values, data were log 2 transformed and quantile normalized [41]. Selection of differentially expressed *m/z* features was performed based on one-way repeated measures ANOVA, using the *limma* package in R [42]. Benjamini-Hochberg false discovery method was used for multiple hypothesis testing corrections at a FDR<0.2 threshold [43]. Visualization of the data, which was based on similarity in expression, was performed using unsupervised two-way hierarchical clustering analysis (HCA) utilizing the *hclust()* function in R to determine the clustering pattern of selected *m/z* features and samples. Principal component analysis (PCA) was performed using the *pca*() function implemented in R package *pcaMethods*.

### 2.6 Pathway enrichment analysis

To evaluate systemic metabolic alterations a metabolome-wide association analysis was performed for discriminatory metabolites at *p* < 0.05 and characterized for pathway enrichment using *mummichog software* [44]. For this analysis, features differing at *p* < 0.05 were selected to protect against type 2 error, and permutation testing (*p* < 0.05) was used in pathway enrichment analysis to protect against type 1 error [45]. Pathways including minimum 5 matched metabolites in total size were selected and annotated using the criteria described below.

### 2.7 Metabolite annotation

Metabolic features were annotated using xMSannotator [46]; confidence scores for annotation by xMSannotator are derived from a multistage clustering algorithm. Identities of selected metabolites were confirmed by co-elution relative to authentic standards and ion dissociation mass spectrometry (Level 1 identification by criteria of Schymanski et al. [47]. Supplemental annotations were made based on high or medium confidence (≥2) with M-H adducts detected in the negative mode. Lower confidence annotations were made using KEGG, (Kyoto Encyclopedia of Genes and Genomes) [48]; HMDB (Human Metabolome Database) [49]; and Lipid Maps [50] databases at 5 ppm tolerance.

### 2.8 xMWAS

Bioenergetic and HRM data from the same set of samples were integrated by using xMWAS based on the sparse partial least-squares (sPLS) regression method for data integration [51]. sPLS is a regression-based modeling approach which performs simultaneous variable selection and data integration, and is designed for problems where the sample size (n) is much smaller than the number of variables (p) and the variables are highly correlated [52]. In addition, the software performs community detection using the multilevel community detection algorithm [53] to identify groups of nodes that are heavily connected with other nodes in the same community, but have sparse connections with the rest of the network. The input for xMWAS included the cellular bioenergetics (13 samples × 6 energetic parameters) and the metabolome (13 samples × 2705 metabolic features which had been quantile normalized and log-transformed) data matrices. Thresholds for determining significant associations must have met the correlation threshold criteria (|r| > 0.5) and p < 0.05 as determined by Student's t-test.

### 2.9 Statistical analysis

The data reported in the metabolomics analyses are derived from platelets isolated from 11 to 13 different donors. Each platelet group was comprised of 3–5 technical replicates, and the data is presented as mean ± SEM. Statistical significance was determined using either a T-TEST or ANOVA with Tukey's post hoc test for data with more than 2 groups, and p < 0.05 was considered significant. The linear correlation between multiple pairs of bioenergetic parameters were determined using the multivariate function of the JMP statistical program (JMP^®^, Version 13, SAS Institute Inc., Cary, NC). A correlations (r-values) table that summarizes the strength of the linear relationships between each pair of bioenergetic parameters and a table with corresponding p-values were generated to identify significant dependencies between parameters. An r-value ≥0.4 with a p ≤ 0.01 are considered significant.

## 3 Results

### 3.1 Platelet bioenergetics and mitochondrial function in healthy subjects

**Fig. 1:**
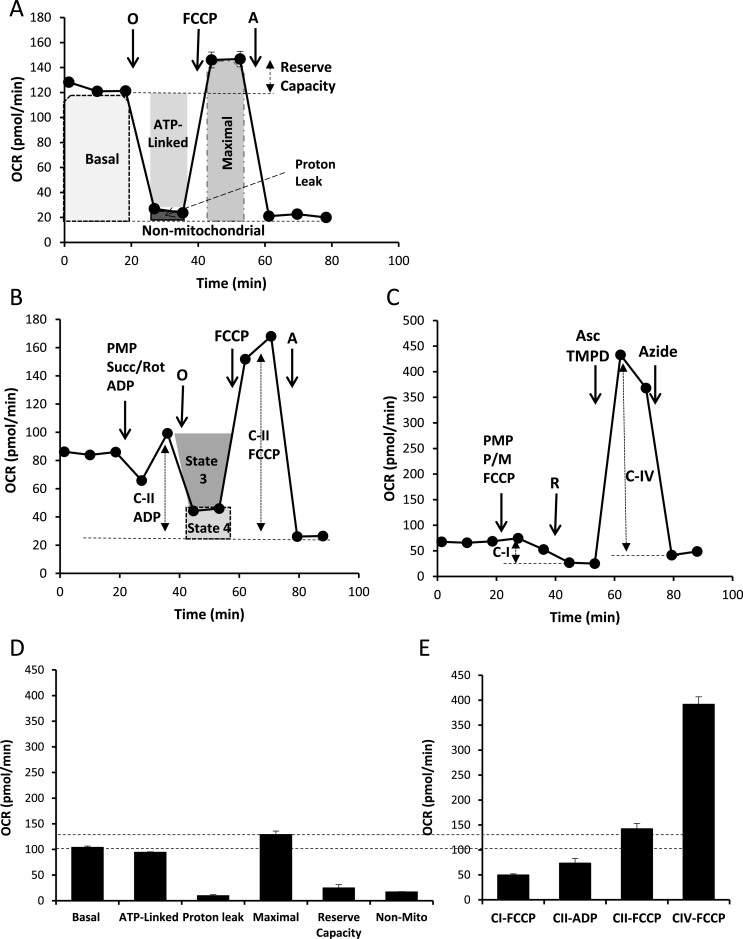
**Defining cellular bioenergetics and mitochondrial function in human platelets. (A)** The mitochondrial stress test (MST) is shown for a typical platelet sample from a healthy donor with the sequential addition of mitochondrial inhibitors (oligomycin, FCCP, and antimycin. The measurement of Basal, ATP-linked, proton leak, maximal, reserve capacity and non-mitochondrial OCR are shown Mitochondrial complex activities were determined in permeabilized cells by providing an excess of substrates. **(B)** After establishing a steady state of oxygen consumption, PMP (plasma membrane permeabilizer), succinate, rotenone, and ADP were injected to stimulate CII activity to couple to ATP production, then oligomycin, FCCP, and antimycin to completely inhibit mitochondrial respiration. State 3 and state 4 respiration of cells in the presence of succinate/ADP followed by oligomycin allows measurement of both complex II and the respiratory controls ratio (RCR). **(C)** PMP, pyruvate, malate, and FCCP were injected to stimulate maximal respiration through complex I. This is followed by rotenone to inhibit complex I and then ascorbate/TMPD to assess the activity of Complex IV, then azide as an inhibitor of complex IV. This figure shows the assay design which allows for the measurement of cellular energetics and mitochondrial respiratory complexes from the same platelet sample. **(D)** Bioenergetic parameters calculated from the MST, and **(E)** Calculation of C-I, CII, and CIV with FCCP for maximal activity and C-II with ADP. Data is shown for technical replicates of 3–5 wells as mean ± S.E.M. for each platelet preparation.

Platelets isolated from 85 healthy volunteers (demographics in Table 1a, Table 1ba,b) were analyzed for the MST and mitochondrial function as we described previously [14,16,19,28]. Fig. 1A–C shows examples of these assays for an individual donor and the calculation of the energetic parameters associated with MST and assessment of oxidative phosphorylation in intact and permeabilized platelets. This approach allows the measurement of bioenergetics from intact platelets and the activity of the key components of oxidative phosphorylation in parallel samples from the same donor [28]. The age range in this healthy population was 18–64 (Table 1a). To determine whether in this cohort age had any impact on bioenergetic or mitochondrial parameters a regression analysis was performed (Table 1b). As can be seen no correlations were observed for any of the parameters and age but this does not preclude the possibility that in older age groups a decrease in bioenergetics occurs as has been reported for blood mononuclear cells in older adults [54]. We also examined the effects of gender and found no significant differences (result not shown). The study is not sufficiently powered at this stage to determine differences in bioenergetics between Caucasian and African American healthy subjects.

**Fig. 2:**
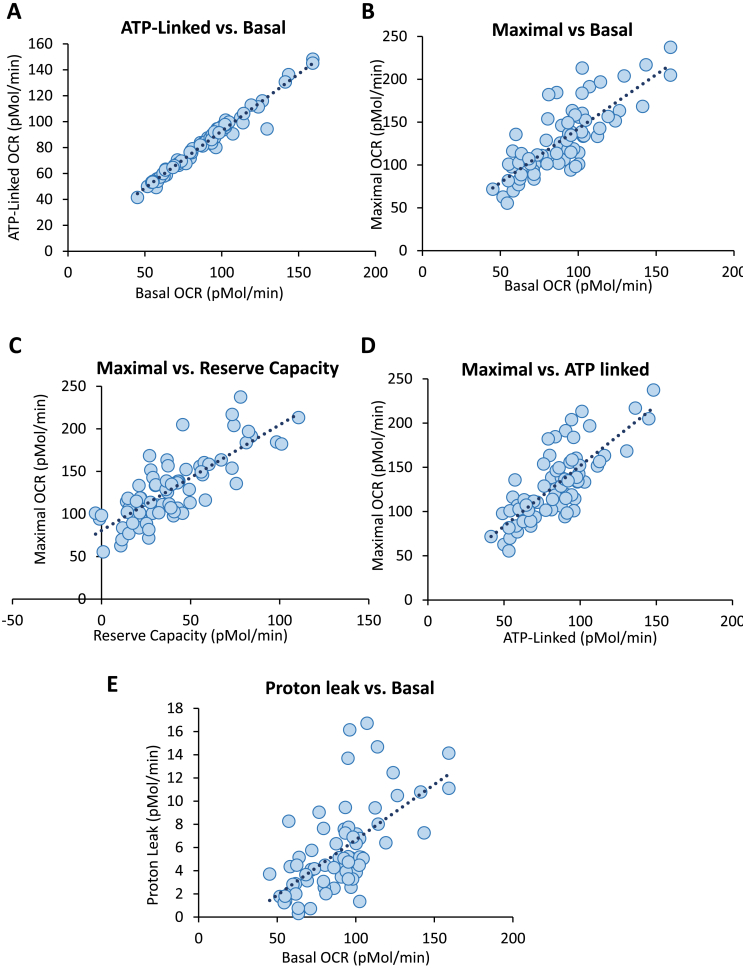
**Relationships between bioenergetic parameters from the mitochondrial stress test (MST) and Oxidative Phosphorylation Assays.** Using the data shown in Fig. 1 multivariate analysis was performed using the JMP statistical package to assess relationships between parameters from the MST and oxidative phosphorylation between healthy platelet donors. Each symbol (n = 85) represents the mean of data from a single individual (3–5 technical replicates) and the dotted line represents the regression. The r-values shown and the results of the significance tests for these data are reported in Table 2.

To establish the relationships between parameters from the MST and activity of mitochondrial complexes in the same platelets, we collected data from the healthy subjects as shown in Fig. 1A–C and subjected them to a multivariate analysis as reported in Table 2 and graphically in Fig. 2. Applying a minimum threshold r-value of 0.4 and p < 0.01, highly significant relationships were evident between basal OCR and ATP-linked OCR, proton leak, and maximal OCR, but not reserve capacity and non-mitochondrial OCR. ATP-linked OCR was positively correlated with maximal OCR but more weakly with proton leak. Reserve capacity was positively correlated only with maximal respiration. Mitochondrial Complex activities were not strongly correlated with each other with the exception of Complex II/FCCP vs Complex II/ADP and a weak association of Complex I and IV. The respiratory control ratio (RCR) is frequently used as a measure of mitochondrial efficiency and in these protocols is measured with complex II-linked substrates as the ratio of activity in the presence of ADP (complex V dependent) or the uncoupler FCCP (ATP synthase independent). The values for RCR were positively correlated with each other consistent with ATP synthase activity placing a limit on the maximal flux through mitochondrial electron transport in coupled mitochondria. Interestingly, the RCR measured with complex II-linked substrates with ADP was negatively correlated with basal, proton leak and maximal OCR.

We reasoned that the lack of correlation of mitochondrial parameters with the intact platelet bioenergetics could arise if mitochondrial capacity is in excess of bioenergetic demand in the intact platelet. To test this we show the comparison of the maximal OCR values for complexes I, II and IV compared to the basal and maximal OCR in the same platelets from the MST (Fig. 1D–E). As we suspected the sum of the maximal activity of complexes I, II and IV are well in excess of the mitochondrial oxygen consumption needed to sustain energy demand in the normal intact platelet.

### 3.2 Relationships between MST parameters in the intact platelet in healthy subjects and patients with SCD

**Fig. 3:**
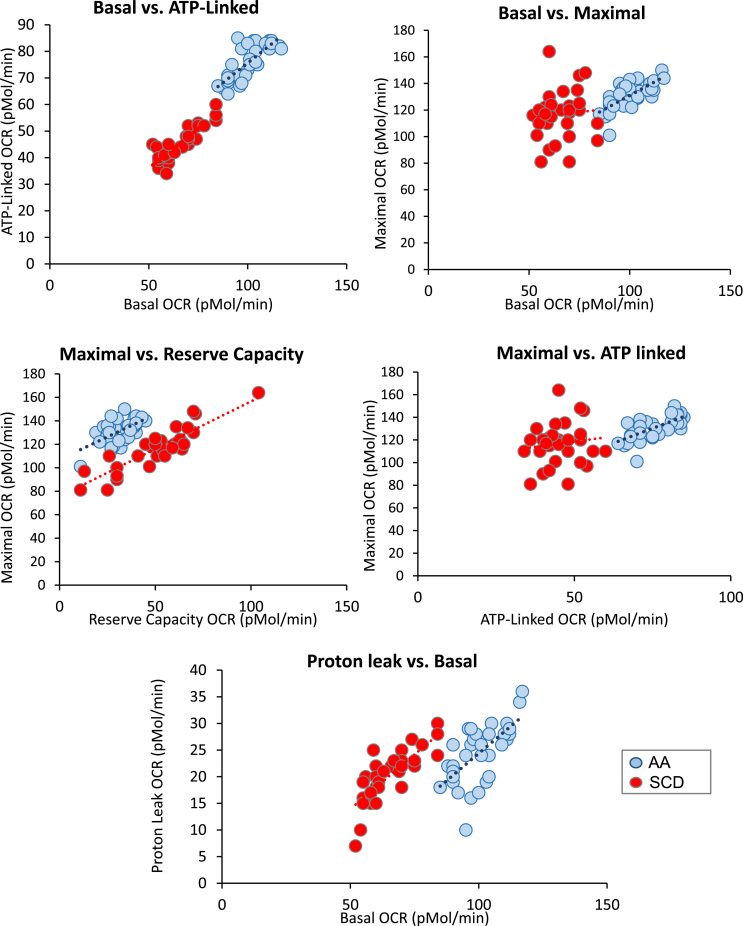
**Relationships between bioenergetic parameters from the mitochondrial stress test (MST) for Healthy African American Subjects and Patients with Sickle Cell disease.** Using the MST assay shown in Fig. 1 multi-variant analysis was performed using the JMP statistical package to assess relationships between parameters from the MST and in African American (AA) healthy subjects (n = 35) and those with SCD (n = 35). Each symbol (blue dots = AA and red dot = SCD) represents the mean of data from a single individual (3–5 technical replicates) and the dotted line represents the regression. The r-values shown and the results of the significance tests for these data are reported in Table 3.

The analysis of the MST test in healthy subjects revealed a number of strong associations between key bioenergetic parameters such ATP-linked respiration with basal and maximal (Fig. 2, Table 2). Using data from an established cohort of SCD patients [14] we hypothesized that the relationships between the MST parameters would be compromised. The demographics for this patient population is reported in Table 1c. The result of this analysis is shown in Fig. 3 and Table 3. Applying a minimum threshold r-value of 0.4 and p < 0.01, highly significant relationships in the African American population were evident between Basal OCR and ATP-linked OCR, proton leak, and maximal. In this cohort, strong correlations between maximal, reserve capacity and non-mitochondrial OCR were also noted. ATP-linked OCR was positively correlated with maximal OCR but more weakly with proton leak. In the SCD patients, the relationship with maximal OCR and basal and ATP-linked OCR was no longer evident. In addition, the correlation between maximal and non-mitochondrial OCR evident in the African American healthy subjects was also lost.

**Table 1c:** Influence of Age on mitochondrial parameters.

	Correlations	Correlation Probability
Basal	0.1948	0.2284
ATP-Linked	0.1944	0.2295
Proton Leak	0.237	0.1408
Maximal	0.181	0.2638
Reserve Capacity	0.0898	0.5816
Non-Mitochondrial	−0.1406	0.387
BHI	0.0578	0.723
Basal ECAR	0.1378	0.3963
Glycolytic Reserve	0.065	0.6903
Complex I	−0.1696	0.2954
Complex II-ADP	−0.0002	0.9989
Complex II-FCCP	−0.06	0.7132
Complex IV	0.0283	0.8622
RCR-ADP	−0.0508	0.7555
RCR-FCCP	−0.0917	0.5737

The relationship between the mitochondrial parameters were analyzed by correlation studies (n = 70–85) using JMP 13 statistical software. Correlation probability of <0.05 were considered significant.

**Table 2:** Correlation between Platelet Bioenergetic parameters.

	Basal	AL	PL	Max.	RC	NM	Comp I	Comp II-A	Comp II-F	Comp IV	RCR-A
Basal											
AL	**0.982**										
PL	**0.623**	**0.514**									
Maximal	**0.793**	**0.771**	**0.466**								
RC	0.255	0.239	0.104	**0.791**							
NM	−0.103	−0.070	−0.272	−0.071	−0.004						
Comp I	0.187	0.151	0.217	0.243	0.197	0.115					
Comp II-A	0.032	0.077	−0.049	−0.034	−0.076	**0.512**	0.228				
Comp II-F	−0.019	0.046	−0.142	−0.014	−0.007	0.389	0.351	**0.849**			
Comp IV	0.221	0.187	0.316	0.359	0.346	−0.046	**0.456**	0.036	0.216		
RCR-A	−**0.433**	−0.362	**−0.417**	**−0.420**	−0.244	0.394	−0.206	**0.594**	0.393	−0.295	
RCR-F	−0.238	−0.191	−0.299	−0.187	−0.068	0.0002	−0.055	−0.284	0.069	-.0771	**0.859**
**Correlation Probability**											
Basal											
AL	**<.0001**										
PL	**<.0001**	**0.0006**									
Maximal	**<.0001**	**<.0001**	**0.0021**								
RC	0.1070	0.1321	0.5163	**<.0001**							
NM	0.5203	0.6627	0.0858	0.6616	0.9786						
Comp I	0.2406	0.3466	0.1722	0.1262	0.2174	0.4751					
Comp II-A	0.8415	0.6341	0.7632	0.8339	0.6349	**0.0006**	0.1513				
Comp II-F	0.9059	0.7765	0.3756	0.9311	0.9635	0.0119	0.0246	**<.0001**			
Comp IV	0.1646	0.2410	0.0441	0.0212	0.0269	0.7745	**0.0028**	0.8231	0.1757		
RCR-ADP	**0.0047**	0.0202	**0.0067**	**0.0063**	0.1239	0.0109	0.1962	**<.0001**	0.0111	0.0618	
RCR-FCCP	0.1333	0.2319	0.0572	0.2409	0.6716	0.9990	0.7550	0.0719	0.6701	0.6319	**<.0001**

The top portion of the table contain R-values above for the correlations in each cell. The bottom portion of the table provides the corresponding p-values. Parameters marked in bold met threshold criteria of r-value >0.4 and p < 0.04. Bioenergetic parameters were determined for individual donors as described in Fig. 1. AL (ATP-linked), PL (proton leak), Max. (maximum), RC (reserve capacity), NM (non-mitochondrial).

**Table 3:** Correlation between Platelet Bioenergetic parameters for HV and SCD.

	**Basal**		**AL**		**PL**		**Maximal**		**Res Cap**	
	AA	SCD	AA	SCD	AA	SCD	AA	SCD	AA	SCD
**CORRELATIONS**										
Basal										
AL	**0.7727**	**0.8740**								
PL	**0.6435**	**0.7777**	0.0113	0.3743						
Maximal	**0.6935**	0.0920	**0.6712**	0.1320	0.2830	0.0049				
Res Cap	−0.1982	−0.3951	0.0309	−0.2977	−0.3496	−0.3688	**0.5687**	**0.8784**		
Non-Mito	0.1701	**0.6522**	0.1100	**0.5049**	0.1353	**0.5915**	**0.6069**	0.0938	**0.6314**	−0.2266
**CORRELATION PROBABILITY**										
Basal										
AL	**<0.0001**	**<0.0001**								
PL	**<0.0001**	**<0.0001**	0.9494	0.0292						
Maximal	**<0.0001**	0.6046	**<0.0001**	0.4567	0.1048	0.9782				
Res Cap	0.2611	**0.0207**	0.8622	0.0872	0.0427	0.0318	**0.0004**	**<0.0001**		
Non-Mito	0.3362	**<0.0001**	0.5356	**0.0023**	0.4456	**0.0002**	**0.0001**	0.5979	**<0.0001**	0.1976

### 3.3 Integrating the platelet metabolome with bioenergetics

The data in Fig. 2 showing a several-fold change in the ATP-linked vs basal OCR parameters between individuals suggests that the underlying metabolome should also differ significantly between individuals. To test this we used platelets isolated from healthy individuals and performed both the MST and HRM. The values for the stored platelets were super-imposable on the data from the healthy donor cohort except for proton leak, which was elevated due to increased levels of fatty acids as we have reported previously [28]. The stored platelets show no other metabolic or functional differences compared to those that are freshly isolated and we have previously shown that the increased proton leak was reversed with the addition of albumin to the medium and with no significant effect on other bioenergetic parameters [28]. This suggests the increase in proton leak is reversible and unlikely to significantly affect the overall metabolome. Bioenergetic or metabolomic assessments were not made in the presence of Albumin since this could potentially bind low molecular weight metabolites and prevent detection by mass spectrometry. As a positive control to assess metabolic plasticity in response to metabolic stress, we also treated the same samples with oligomycin, which is an inhibitor of the mitochondrial ATP synthase, and used in the first step of the MST (Fig. 1).

**Fig. 4:**
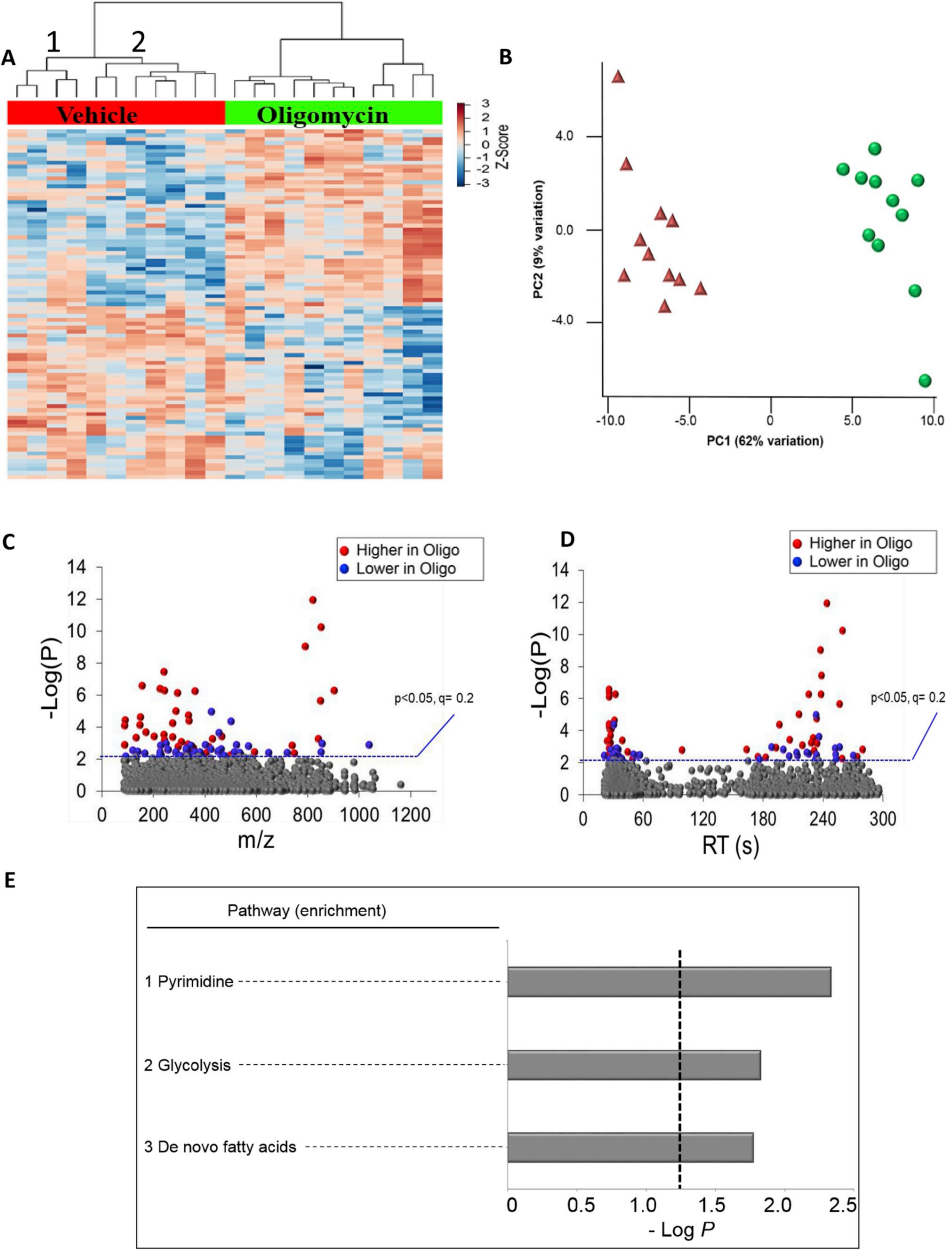
**Metabolic separation of features after Oligomycin exposure in human platelets. (A)** Unsupervised hierarchal clustering heatmap indicates that intensity of 89 features which drive the separation between vehicle control and Oligomycin groups. (**B)** PCA plot showing separation of the vehicle control group (shown in red) and Oligomycin exposed group (shown in green), through the 1st and 2nd principal components. (**C)** Type I Manhattan plot of *m*/*z* features plotted against the –*LogP value.* Shown in gray are the 2572 features identified after filtering and normalization. 89 features were found to be different between the two groups using the criteria (p < 0.05, FDR < 0.2) as indicated by the blue dotted line. Shown in red were features identified to be increased after exposure (45/89) and in blue features which were decreased (44/89). **(D)** Type II Manhattan plot using retention time plotted against –*LogP* value. For a list of annotated features from the pathway enrichment see Supplementary Table 2. (**E)** Pathway enrichment analysis of stored human platelets after Oligomycin exposure compared to vehicle control. A total of 3 enriched pathways were determined (Filled bars indicate significance and the cutoff (p < 0.05) is indicated by the dotted line). For a list of annotated features from the pathway enrichment see Supplementary Table 3.

Over 3500 metabolic features were detected of which 3150 were present in 8 out of 13 donors in control and oligomycin treated samples. To identify the metabolic pathways in these donors, we analyzed 3150 metabolites using the KEGG pathway database (Table 4). The results show that 924 features were matched with KEGG-identified metabolites representing 58 metabolic pathways including arachidonic acid metabolism, glycolysis and fatty acid metabolism, critical for platelet function (Supplementary Table 1). Next, we examined metabolic responses of the platelets to oligomycin treatment. Mass spectral data processing yielded 2572 features in two groups after processing. ANOVA on these features revealed 89 metabolites were changed by oligomycin (p < 0.05 at FDR of 0.2) and are presented by HCA (hierarchical clustering analysis)-heat map (Fig. 4A) and PCA plots (Fig. 4B). Manhattan plots, based upon RT, *m/z* and abundance of metabolites show that of the 89 metabolites, 45 metabolites were higher and 44 metabolites were lower in oligomycin-treated platelets compared to the vehicle group (Fig. 4C and D). Supplementary Table 2 shows detailed information on 34 annotated features. To examine the metabolic pathways associated with 89 metabolites altered by oligomycin, pathway enrichment analysis was performed using *mummichog*. The results showed that the pyrimidine, glycolysis and gluconeogenesis and *de novo* fatty acid pathways were altered by oligomycin treatment (Fig. 4E). The detailed information on metabolites associated with these pathways is provided in Supplementary Table 3.

**Table 4:** Platelet metabolome.

Group	Average No. features per subject ±SEM	Total No. features present in 8/11 donors	Feature hits using KEGG ±5 ppm	Pathway Mapping >4 hits in pathway
Vehicle	3175 ± 25	3150	924	58
Oligomycin	2980 ± 39	3099	940	58

The table represents features that were found for platelets before and after treatment with oligomycin (n = 11).

**Fig. 5:**
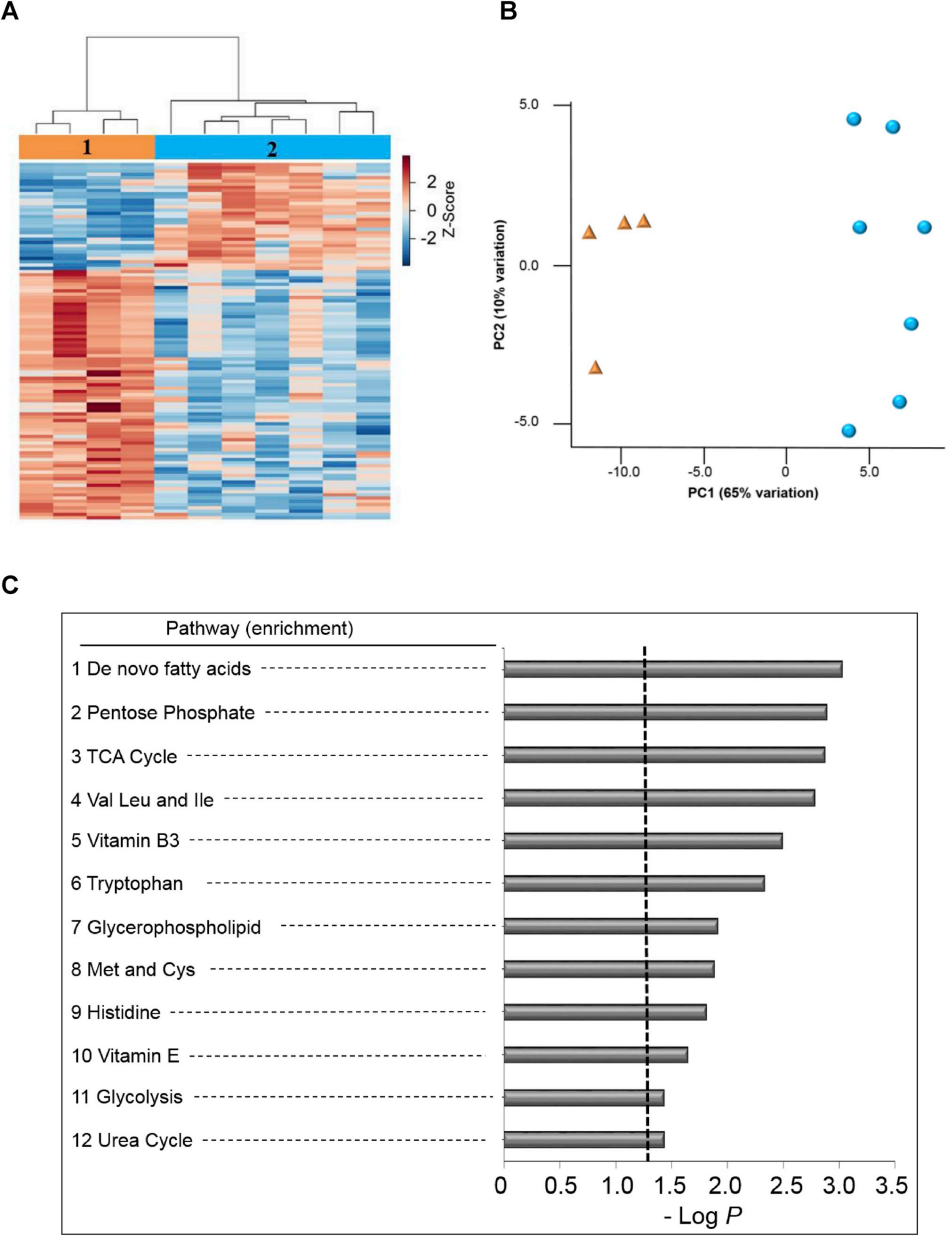
**Metabolic separation of individuals into 2 clusters at baseline. (A).** Unsupervised hierarchical clustering heatmap indicates that intensity of 110 features driving the separation between metabolic groups 1 and 2. **(B).** PCA plot showing separation of the metabolic group 1 (shown in green) and metabolic group 2 group (shown in red), through the 1st (59% variation) and 2nd (11% variation) principal components. For a list of annotated features from the pathway enrichment see Supplementary Table 4. **(C)** Pathway enrichment analysis of stored human platelets at baseline. A total of 12 enriched pathways were determined (Filled bars indicate significance and the cutoff (p < 0.05) is indicated by the dotted line). For a list of annotated features from the pathway enrichment see Supplementary Table 5.

Because the platelets have a broad range of bioenergetic parameters at basal level (Fig. 1, Fig. 2) we examined whether this is also reflected in the metabolome in the vehicle group. The unsupervised HCA-heat map for the metabolome profiles for all 11 donors shows two distinct clusters in the vehicle group (labeled groups 1 and 2 in Fig. 4A). To test whether the two groups differed in their basal metabolism, we analyzed the 2705 processed metabolic features. ANOVA shows that 110 features differed in two vehicle groups as shown in HCA-heat map and PCA plots (Fig. 5A and B). Annotation and the details of these features are provided in Supplementary Table 4. The results of pathway enrichment analysis show that 12 metabolic pathways were different in the two groups, including fatty acid metabolism, pentose phosphate, TCA cycle, branch chain amino acids and vitamin B3 were the 5 metabolic pathways showing the most prominent differences (Fig. 5C, see Supplementary Table 5 for the detailed information of features).

**Fig. 6:**
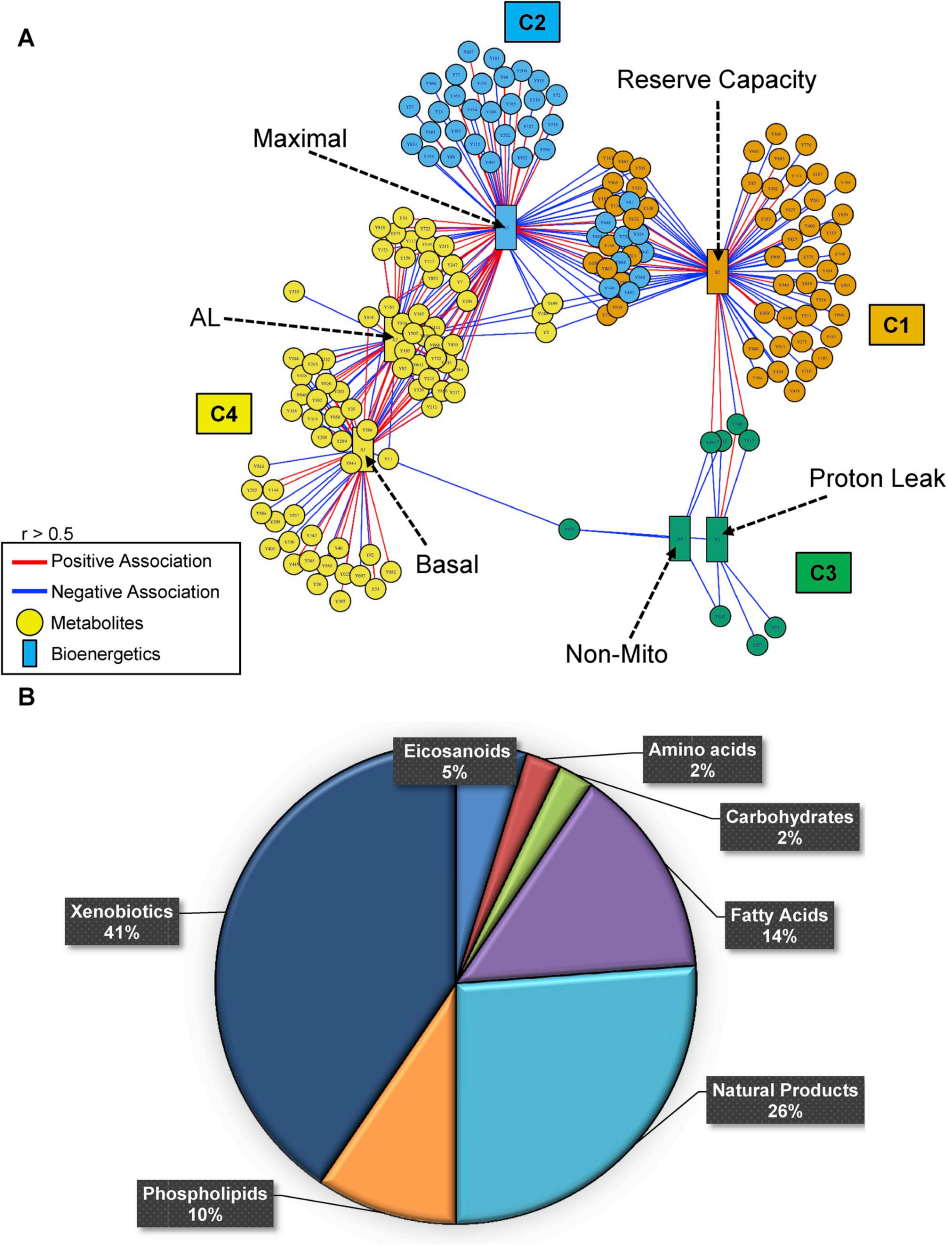
**Association of the metabolome with bioenergetics.** Bioenergetics and HRM data from the same set of samples were integrated by using xMWAS in order to conduct pairwise correlation analysis between the cellular bioenergetics and the metabolome. Four major metabolic communities were detected; community 1 (orange); Reserve capacity was found to be associated with 70 [23] *m*/*z* features; community 2 (blue); Maximal was found to be associated with 91 [23] *m*/*z* features; community 3 (green); proton leak and non-mitochondrial was found to be associated with 8 [3] *m/z* features and; community 4 (yellow); Basal and ATP-Linked OCR were found to be associated with 59 [17] *m/z* features with |r|>0.5 at p < 0.05). See Table 4 and Supplementary Table 6 for the direction of correlations. Red lines indicate positive associations, and blue lines indicate negative associations. The numbers in parenthesis indicate annotated associated features.

Next, to examine the association between the basal metabolome and bioenergetic parameters, we correlated the 6 bioenergetic parameters shown in Fig. 1 with the 2705 metabolic features from the vehicle group using xMWAS [40]. The result of xMWAS analysis yielded 4 distinct communities encompassing over 100 features and 6 bioenergetic parameters with the number of positive and negative associations between metabolites and bioenergetics parameters (r > 0.3–0.5, p < 0.05, Fig. 6, see Table 5 for summary and the details and for annotation details see Supplementary Table 6). Of these metabolites, 42 were further analyzed for annotation and categorized by their function as shown in the pie chart (Fig. 6B). Communities 1 and 2 have the strongest number of interactions with Reserve Capacity and Maximal OCR, whereas community 4 has a stronger influence over ATP-linked and Basal OCR. Community 3 has the smallest number of interactions and is associated with non-mitochondrial OCR and proton leak.

**Table 5:** Correlations Summary from xMWAS.

	Basal	AL	PL	Maximal	RC	NM
	Correlation = 0.3					
Pos. correlation	28	26	1	35	16	0
Neg. correlation	30	29	7	56	55	4
No correlation	116	119	166	83	103	170
Total	174	174	174	174	174	174
	Correlation = 0.4					
Pos. correlation	28	26	1	35	15	0
Neg. correlation	30	29	7	56	54	4
No correlation	116	119	166	83	105	170
Total	174	174	174	174	174	174
	Correlation = 0.5					
Pos. correlation	28	26	1	35	16	0
Neg. correlation	30	29	7	56	54	4
No correlation	116	119	166	83	104	170
Total	174	174	174	174	174	174
	Correlation = 0.6					
Pos. correlation	13	17	0	22	7	0
Neg. correlation	15	14	4	40	37	3
No correlation	88	85	112	54	72	113
Total	116	116	116	116	116	116
	Correlation = 0.7					
Pos. correlation	1	4	0	6	0	0
Neg. correlation	1	3	0	7	9	0
No correlation	19	14	0	8	12	0
Total	21	21	0	21	21	0

The data presented in the above table reflects the total of correlations between metabolic features and bioenergetics parameters. The p-value for this data set is < 0.05 with correlations set from a minimum of 0.3 to a maximum of 0.7. AL (ATP-linked), PL (proton leak), RC (reserve capacity), NM (non-mitochondrial).

## 4 Discussion

It is well recognized that human metabolism varies between individuals and may contribute to the severity or susceptibility to environmental or pathological stress. From a precision medicine perspective, understanding these relationships will be critical in developing personalized approaches to interventions designed to change metabolism appropriately including lifestyle modifications and therapeutics. The challenge is then to develop platforms in which untargeted metabolomics data, as a measure of metabolic responses, can be integrated with measures of bioenergetic performance.

Using a panel of healthy donors, we developed a protocol to measure both the mitochondrial activity of the intact platelet and the performance of the oxidative phosphorylation pathway in the same individual (Fig. 1). This approach allows us to make a quantitative estimate of the variability of metabolic and bioenergetic functions using readily accessible platelet samples as a platform. The MST in combination with the measurement of oxidative phosphorylation in permeabilized platelets generates a number of parameters, which can be directly compared without confounding artifacts introduced by the disruptive procedures used to isolate mitochondria. Maximal respiration is determined in the presence of the uncoupler FCCP and it is then possible that this does not truly represent the maximal capacity due to differences in the sensitivity to the uncoupler between individuals. Although the FCCP concentration was optimized when the methods were established there is not sufficient sample to perform an FCCP dose response for each subject so this remains a possibility. A further constraint on the interpretation of the FCCP-dependent OCR as representing maximal respiration is the possibility that ATP synthase may be limiting. This is not the case for platelets since we have shown that the reserve capacity (maximal-basal OCR) is fully utilized in thrombin activated platelets for the purpose of ATP synthesis [19]. Interestingly, we found that the mitochondrial function required for the intact platelet is substantially below the potential capacity for oxidative phosphorylation evident in the permeabilized platelets (Fig. 1). It follows that the platelet bioenergetic demand is not limited by the capacity of oxidative phosphorylation and in this case, no association of mitochondrial complex activities with intact platelet bioenergetics would be expected.

Since both the genetic and biogenesis programs vary between individuals we reasoned that the need to meet specific energetic demands would result in adaptive metabolic plasticity between individuals. For example, one could predict that differences in mitochondrial efficiency between individuals would result in varying proportions of the oxygen consumption being directed towards ATP synthesis when individuals are compared. Using a multi-variate analysis we confirm that the ability of platelets to meet bioenergetic demand through oxidative phosphorylation varies substantially between individuals (Fig. 2). The plot of ATP-linked OCR vs basal OCR shows a remarkably tight correlation consistent with this wide range of plasticity in human metabolism in normal resting platelets. This ATP-linked OCR makes a substantial contribution to the Basal OCR so this relationship might be expected on this basis alone. However, Proton leak-dependent OCR can be measured with the same precision as ATP linked-OCR but is much more weakly correlated suggesting that alternative interpretation that these bioenergetic parameters are independently regulated. The finding that maximal respiration is well correlated with reserve capacity is expected since this parameter is derived from subtracting the basal OCR from the maximal. Interestingly, maximal OCR is strongly correlated with ATP-linked OCR but only weakly with proton leak suggesting that changes in mitochondrial efficiency between individuals are not strongly influenced by proton leak in normal subjects. The negative correlation of RCR with proton leak and maximal respiration suggests that as RCR increases (equivalent to increased efficiency) maximal respiration and proton leak decrease so allowing energy demand to match overall capacity. This is also consistent with the strong correlation of maximal respiration with ATP-linked OCR. Non-mitochondrial OCR is measured after the addition of antimycin A and shows a positive correlation with Complex II in the presence of ADP but not FCCP or other mitochondrial complexes. This is particularly interesting as reverse electron transport from Complex II through complex I is currently of great interest and could be contributing to the antimycin A-insensitive OCR [11].

These data strongly support the concept that the bioenergetic program in human subjects varies between individuals depending on genetic, dietary and environmental factors. The program is plastic and adaptable to meet the energetic demands of a particular cell type in both quiescent conditions and during biological activation. For example, platelets show a 2–3 fold variation in the amount of oxygen needed to maintain ATP (Fig. 2) and both glycolysis and oxidative phosphorylation contribute to the energy demands of platelet aggregation(19,20). This concept suggests that under pathological conditions in which metabolism or bioenergetics are targeted these relationships may break down. We tested this hypothesis in a cohort of African Americans with SCD which we have previously shown have a bioenergetic defect in mitochondrial ATP synthase [14]. We found that the strong relationship between ATP-linked and basal OCR was maintained although the dynamic range for the ATP-linked OCR was significantly depressed as we have reported previously [14]. In the healthy subject cohort, there are significant relationships between the parameters which contribute to basal respiration (ATP, proton leak) and maximal which are lost in the SCD patients (Fig. 3, Table 3). This suggests that the program, which links maximal respiratory capacity with other respiratory parameters, is no longer functional and will then constrain the overall bioenergetic plasticity for the SCD patients. Non-mitochondrial respiration is calculated as the OCR that remains after treatment with an inhibitor of mitochondrial electron transport. What contributes to non-mitochondrial OCR healthy subjects is not clear but in the SCD patients, it has been shown that xanthine oxidase is making an important contribution [14]. In African American healthy subjects, the non-mitochondrial OCR is positively correlated with maximal respiration and this relationship is lost in the SCD patients. Clearly, further studies are needed to determine how this relationship is established in healthy platelets and whether xanthine oxidase is playing a role.

Using unsupervised HCA-heat map analysis, vehicle and oligomycin-treated platelets resulted in a metabolism-specific pattern (Fig. 4A). The mechanism of oligomycin is to inhibit ATP synthesis and inhibition of ATP stimulates glycolysis in platelets [19], and indeed KEGG and pathway analysis indicated that among the major pathways glycolysis and gluconeogenesis are changing in response to oligomycin (Fig. 4E, Supplementary Table 3). Other metabolic pathways, which are showing differences, are also consistent with a switch to a more glycolytic phenotype including changes in fatty acid and pyrimidine metabolism. In addition, several metabolites involved in *de novo* fatty acid metabolism were decreased Fig. 4E and Supplementary Table 3. Pyrimidine metabolism was another pathway altered by decreased mitochondrial function. Mitochondrial dysfunction is known to affect nucleotide metabolism [55].

In analyzing the metabolomics data further, we found that an unsupervised clustering of the untargeted metabolome of platelet donors identified 2 metabolically distinct groups. We hypothesized that segregation of the platelets from individuals into groups 1 and 2, revealed after treatment with oligomycin, would be also correlated with parameters from the MST. To test this hypothesis, the relative amounts of the metabolites identified in the metabolome were correlated with parameters from the MST. Using an approach of unsupervised network integration and clustering of the bioenergetic phenotype with metabolomics, we visualized interactions between these two variables and identified key modulators. As shown in Fig. 4, Fig. 6 communities and the strength of relationships between the MST parameters established independently from the multi-variate analysis in Fig. 2, Table 2, are recapitulated on the basis of the metabolome in the resting platelet. For example, in the xMWAS, the proximity of the communities is proportional to the closeness of the relationships. Proton leak, ATP-linked and Basal OCR are all well correlated and are clustered whereas reserve capacity is distant from these parameters and is linked only through maximal respiration. From the xMWAS correlation summary in Table 4, it is clear that there are both positive and negative correlations with metabolic features and the MST parameters. This gives confidence in the validity of the data set since one would expect that in comparing the metabolome between different individuals that as one pathway decreases in relative activity (e.g. OXPHOS) then another (e.g. glycolysis) may increase. The visual depiction of the data in Fig. 6A also illustrates that the MST parameters have numerous metabolites that are modulated independently from each other. Fig. 6B shows the assignment of features identified as correlating with xMWAS data into different chemical classes. As expected fatty acids, amino acids and carbohydrates are present. Interestingly, approximately 41% of the metabolites can be characterized as xenobiotics being derived from the environment or pollutants. These findings are important since they support the concept that exposure to environmental pollutants impact bioenergetic function [56].

Taken together these data demonstrate how the metabolome in the intact platelet is functionally integrated with bioenergetics. An important result is that the associations between parameters in the MST are well correlated with nearly 200 metabolites measured in the resting platelet. This suggests that selection of an appropriate stressor for a defined pathological condition can be used *in vitro* to identify a metabolome associated with increased susceptibility to disease. An intriguing aspect of this data is the finding that xenobiotics are correlated with platelet bioenergetic parameters. This raises the interesting possibility that platelets energetics and metabolomics could be used to assess the susceptibility of a population to environmental exposure and the severity of the toxic response among individuals. This has important implications for the application of precision medicine to metabolically related disease including Alzheimers and other age-related pathologies. Clearly, the platelet is a powerful platform for the linking disease mechanism to metabolomic profiling.
